# A visualization-supported, hierarchical, action-learning model for driving behavior in a V2X environment

**DOI:** 10.1371/journal.pone.0336268

**Published:** 2026-01-02

**Authors:** Xuantong Wang, Jing Li, Jecca Bowen

**Affiliations:** 1 Texas Tech University, Lubbock, Texas, United States of America; 2 University of Denver, Denver, Colorado, United States of America; Beijing Institute of Technology, CHINA

## Abstract

Understanding human driving decisions is crucial for intelligent transportation research. Most existing studies focus on individual vehicles in limited contexts, which restricts broader applicability of results. Leveraging Vehicle-to-Everything (V2X) infrastructure, this study introduces a machine learning framework to model driving actions and detect outliers across diverse environments. This approach features a semantically enabled clustering method that groups similar driving behaviors based on speed and actions. It also adds a time-series learning model to identify typical driving behaviors across various contexts, thereby enabling detection of abnormal driving actions. A suite of visual tools has been developed to help interpret driving patterns, and a case study using six months of data from a V2X pilot project in Tampa, Florida, demonstrates the framework’s effectiveness in modeling human driving decisions. It also highlights discrepancies between context-appropriate driving behaviors and actual human actions to improve safety and efficiency for transportation planners and individual drivers.

## 1. Introduction

Vehicle-to-Everything (V2X) communication technology provides a rich repository of data on driver behaviors, including vehicle speed, acceleration, and braking [1] as well as environmental context variables, such as road conditions and traffic flow. These large-scale, real-time, V2X data from multiple vehicles offer the most comprehensive view to date of real-world traffic dynamics and human responses under diverse environmental conditions, effectively overcoming the data availability challenges that constrained earlier research efforts and supporting investigations into risky driving, transportation safety analysis, traffic management, and the development of intelligent transportation systems [2,3]. With this valuable information available, it becomes possible to answer two key research questions: How can we detect normal and abnormal driving actions in diverse driving scenarios? Then, once these patterns are understood, how can these results be communicated in a way that makes them clear and useful to a wide range of stakeholders in order to enhance safety and improve decision-making in the realm of traffic management and policy [4,5].

Most existing methods for modelling driving decisions have yet to maximize the analytical potential of V2X datasets due to the inherent complexity of capturing diverse driving contexts, external environmental impacts, and behavioral variations among drivers. In light of this need, we propose a holistic approach to modeling and detecting typical driving decisions across diverse contexts. The core component of our methodology is a multi-step clustering method, strategically deployed to identify and categorize similar driving contexts and styles. In the first stage, trajectories are grouped based on driving speed. In the second stage, a temporal, hierarchical learning model leveraging a Long Short-Term Memory (LSTM) model discerns typical actions in comparable driving contexts. To enhance interpretability of the model results and driving behaviors, a suite of data visualization tools has been integrated, which enables thorough exploration of the complex patterns and relationships inherent in driving decision data and enhances the model’s explainability, interpretability, and transparency [6]. Together, these components form an effective framework for assessing driving decisions across various transportation contexts, providing a comprehensive and insightful approach to the study of driving behavior. To demonstrate the efficacy of our framework, our study leverages an extensive, six-month Basic Safety Message (BSM) dataset [7] collected from a diverse fleet of vehicles that participated in a pilot V2X project in Tampa, Florida.

Our work contributes to the field of driving safety in several ways. First, we analyze driving behaviors using a pilot V2X dataset, offering a more representative model of real-world driving conditions compared to confined simulations or limited study domains. Second, our semantically enabled clustering method offers an effective way to identify natural groupings in this real-world driving data based on driving action characteristics, followed by a hierarchical action determination process that successfully identifies typical and atypical actions within those initial groups. Third, our visual analytical tools, such as attribute visualizations and time-sequence plots, support the interpretability and explainability of complex driving patterns and relationships within the traffic data. Collectively, these contributions advance the field of driving behavior analysis by leveraging V2X data and providing a more robust and evidence-based framework for understanding the interplay of various driving actions and risks.

## 2. Related work

The ability to accurately model and understand driving decisions is critical for the field of transportation management, with far-reaching implications for enhancing road safety, optimizing vehicle performance, and developing effective driver training programs Modeling driving decisions inclue predicting how drivers perceive, decide, and act in varied conditions [8]. Traditional data sources such as reports, experiments, and simulations often overlook real-world complexity, limiting their practical use in driving and transportation planning [9–11]. To address these limitations, some researchers have used computer vision to collect and process real-time traffic data from in-vehicle or roadside cameras However, this approach can raise privacy concerns, be costly, and may not efficiently deliver real-time safety information to drivers [12].

Therefore, there is a growing need for efficient, accessible, reliable, and comprehensive real-world datasets—such as those collected by Vehicle-to-Everything (V2X) systems—to support effective management, prompt recommendations for drivers, and enhanced transportation safety. Because V2X systems integrate data about many important contextual variables from various connected devices, the resulting datasets provide a much more comprehensive view of driving actions than previous data collection methods, enabling nuanced assessment of interactions between vehicles, drivers, and infrastructure while also supporting more accurate predictive modeling with real-time data. The advancement of V2X technology, particularly with the integration of modern communication protocols such as 5G and Cellular-V2X (C-V2X) [13], supports a real-time, comprehensive ecosystem that significantly enhances traffic management and safety, while also improving vehicle performance under different conditions [14,15]. This system facilitates the rich exchange of sensor data between vehicles (V2V) and infrastructure (V2I), enabling dynamic traffic control based on real-time vehicle flow to reduce congestion and improve efficiency. Furthermore, V2X enables cooperative driving applications—such as platooning and intersection movement assistance—that help vehicles navigate more efficiently. For safety, V2X provides vehicles with enhanced situational awareness that extends far beyond a driver’s line of sight, enabling advanced collision avoidance and proactive safety measures. Vehicles can receive immediate warnings from other connected cars and infrastructure about hazards like sudden braking. Additionally, Vehicle-to-Pedestrian (V2P) communication alerts drivers to the presence of vulnerable road users, which can significantly reduce collision risks. Therefore, the deployment of V2X technology provides a powerful foundation for a smarter, safer, and more efficient transportation system. Despite their potential, V2X data remain underutilized in driving decision models due to limited deployment, inconsistent data standards, and frequent technology updates. The large volume of data also poses challenges for extracting meaningful insights. While we are unable to address large-scale deployment factors, our current study makes progress on the third challenge by developing effective and replicable strategies for analysing, interpreting, and applying insights from these large datasets.

In addition to these diverse traffic data sources, many different approaches and tools for modeling driving actions and patterns have been proposed [16]. For example, Markov models [17], support vector machines [18], and neural networks [19] have been widely used to predict driver actions. Additionally, dynamic Bayesian networks [20], Gaussian processes [21,22], and inverse reinforcement learning [23] serve as tools for modeling driver actions. However, there is a challenge in building a generalizable model that can be adapted across different driving scenarios, rather than being limited to specific situations, such as car-following [24]. In recent years, many researchers have utilized deep learning models to address this deficiency. For example, imitation learning has gained prominence as a method for understanding the decision-making processes of human drivers by analyzing observed trajectories and actions. Additionally, behavioral cloning [25] has been used as a direct approach to learning from observational data. Generative models [26] like the Generative Adversarial Networks (GAN) show promising results for modeling human driving behavior based on various spatiotemporal and behavioral contexts [27]. For example, GAN-based approaches have been applied to support scene understanding [28] and to produce synthetic data for predicting vehicle accident risk levels [29]. Despite these advancements, these learning methods exhibit similar limitations to those of traditional modeling approaches, including being conducted in limited naturalistic driving environments [30].

To address these challenges, we leverage extensive V2X data [31] to examine driving actions. We use a two-step clustering method to group vehicle trajectories by speed and actions, followed by an LSTM model to identify common behaviors within each driving context. This approach effectively captures driving patterns while avoiding the training complexities of Reinforcement Learning, providing deeper insights for researchers, transportation planners, and drivers.

## 3. Method

### 3.1 Models

#### 3.1.1 Overall process.

**Fig 1:**
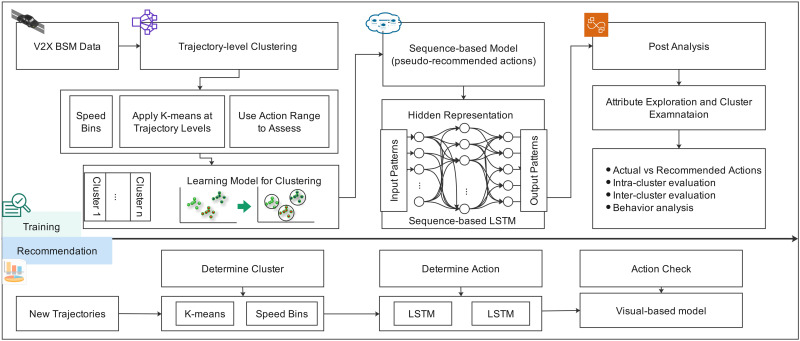
Clustering workflow.

The model objectives are (1) to determine the next action (referred to here as the “recommended action”) based on a given driving context and known, typical actions for similar contexts and (2) to assess whether the actual action deviates from the recommended action. To accomplish this, the following steps are implemented (see Fig 1):

Step 1: Clustering is performed at the trajectory level using average speed, average acceleration, and average yaw rate. The upper and lower bounds of the typical ranges for each action are derived from the trajectories in each cluster.
Step 2: The learning model learns patterns in actions based on trajectories in the same cluster, producing pseudo-recommended actions for subsequent comparison. Here we use a sequence-based LSTM model, in which actions are analyzed at every time step.
Step 3: Post-analysis evaluates the deviation of actual actions from the model’s recommended actions, providing insights into the model’s performance and areas for potential improvement.

During the training phase, an extensive dataset is employed to train the model, allowing it to learn and adapt to various driving contexts. In the recommendation phase, the trained model is applied to new trajectories, which are sorted into clusters based on the driving patterns and behaviors that the model learned during the training phase. A suite of data visualization techniques is then employed to provide insights into the model output, including patterns, behaviors, and outliers within and across clusters. The process of data preparation and trajectory reconstruction is described in S1 Fig. The formal definition of the learning problem is in S1 Text.

#### 3.1.2 Trajectory-level clustering.

In the model’s first clustering procedure, road speed is a key factor, and trajectories are organized into distinct bins according to their average speed. We divided the vehicle speed into three distinct bins including low, medium, and high speeds based on established traffic flow definitions and prior research on GPS trajectories [32–35]. This approach ensures replicability and semantic interpretability of the bins. Each bin represents a range of average speeds, and trajectories are grouped with similar speed profiles. Within each bin, K-means clustering is applied to further categorize the trajectories based on other action attributes, including average acceleration and yaw rates. The definitions of clustering are described in S2 Text. This generates distinct, hierarchical cluster labels, which offer a systematic way to categorize trajectories based on their unique characteristics. These clusters will set the foundation for subsequent phases of the analysis and contribute to the overall comprehension of driving patterns and decision-making within the dataset.

#### 3.1.3 LSTM model for action determination.

**Fig 2:**
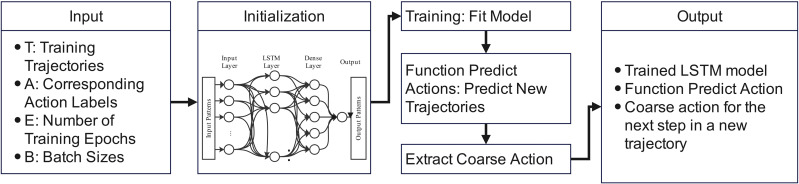
LSTM workflow.

In the next phase, these clustered training data become the input for an LSTM model, which observes patterns in the data of observed trajectories and driving contexts, applies these patterns to a new trajectory not included in the training data, and outputs a recommended driving action. To determine the recommended action, the LSTM model is applied to each cluster (speed-based clusters and action-based sub-clusters within each speed bin) separately, incorporating a state-action pair using a stateful LSTM architecture. This allows the model to retain information across time steps within each cluster, meaning that the hidden state of the LSTM is preserved from one time step to the next, and the model can capture and remember sequential patterns within the trajectories. For each cluster, the LSTM model processes state-action pairs by learning the temporal dependencies and patterns specific to that cluster. This process is based on the assessment of the mean squared error (see S3 Text). This approach (Fig 2) ensures that the model adapts to the characteristics of each subset of trajectories, providing a nuanced understanding of the driving decisions made within particular driving contexts.

#### 3.1.4 Visual-based action analysis.

Our visual-based action analysis offers a comprehensive approach to gaining insights into the clusters and recommendations made by the model. This section includes various visualizations and analyses that facilitate our understanding, interpretation, and assessment of the model’s performance. Visualization allows us to observe how different attributes—such as speed, position, and acceleration—influence driving behaviors and decision-making. By visually representing the model’s outputs, we aim to improve explainability, making it easier to interpret how certain variables contribute to the model’s driving action recommendations. Additionally, visualizing the clustering results makes it easier to understand the various groupings, identify patterns, and validate the significance of the clusters. These visual tools provide a deeper look at both the model’s accuracy and the driving factors behind its predictions, making the analysis more transparent and interpretable. We demonstrate this visual-based analysis with the examples below.

a Cluster profile visualization

**Fig 3:**
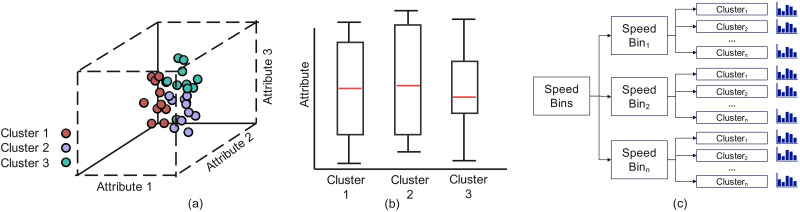
(a) Distribution of trajectories assigned to different clusters, (b) corresponding boxplots showing the attribute distributions within each cluster, and (c) a tree plot showing the distribution of attributes both between and within clusters.

The purpose of cluster profile visualization is to provide a high-level, visual summary of attribute distributions for each cluster (Fig 3), which can reveal patterns and variations in driving behavior. As mentioned above, in our proposed method, vehicle data are first clustered based on average speed and segmented into low-, medium-, and high-speed bins. Further clustering techniques are then applied within each speed bin in order to group trajectories with similar acceleration and yaw rates. To visually represent the distribution of key attributes within each cluster, boxplots are used to demonstrate the central tendency, variability, and outliers. By analyzing these cluster profiles through boxplots, we can identify characteristic driving patterns, such as safe driving with moderate speeds and smooth deceleration, as well as abnormal driving behaviors, such as aggressive driving with high speeds and sharp braking. This approach helps identify common driving behaviors, variations, and safety risks, allowing for better understanding of driving dynamics and possible targeted interventions in driver behaviors and safety.

Visualizing data distribution, especially the relevant attributes involved in the modeling process, is essential for assessing driver behaviors and evaluating model performance. To achieve this, we use a 3D diagram to analyze attribute distribution (Fig 3a), making it easy to identify clusters and outliers. Additionally, we generate separate bar plots (Fig 3b) for each cluster to provide a detailed view of recommendation errors relative to specific attributes. This approach highlights where the model performs well and where it faces challenges while simultaneously shedding light on the causes of recommendation errors, particularly those related to variations in driving behaviors. By examining how different attribute combinations—such as speed, acceleration, and braking patterns—impact recommendation accuracy, we can pinpoint clusters that (1) demonstrate similar behaviors; (2) reveal where the model struggles with complex or inconsistent driving behaviors, such as aggressive driving or frequent, abrupt maneuvers; and (3) contain outliers. This can all be achieved through a hierarchical view based on a tree plot of speed bins, clusters within speed bins, and attribute distributions within each cluster (Fig 3c). These insights deepen our understanding of the relationship between driving patterns and model recommendation errors, revealing areas for targeted improvement.

b Evaluation and time-series view of action and recommendation

**Fig 4:**
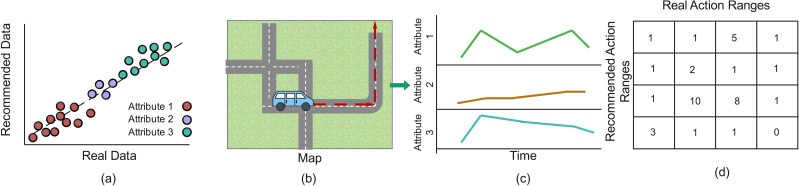
(a) Mean absolute error (MAE) per cluster based on recommended and actual actions, with trajectory-level results illustrated through (b) a map-based view showing locations of trajectories with high or low MAE performance, (c) attribute profile plots, and (d) a confusion matrix for actual and recommended actions.

To assess the model’s performance and improve its explainability, it is essential to compare recommended and actual outcomes (Fig 4a) and to visualize the spatial locations of trajectories on a map (Fig 4b)—particularly those trajectories that performed well or poorly based on the proposed clustering. In many machine learning studies, a key metric for evaluating model performance is mean absolute error (MAE), which measures the difference between predicted and actual values (see S3 Text). By calculating the MAE for each individual cluster, we gain a more detailed understanding of the model’s accuracy within specific data groups. However, in our proposed work, we aim to detect variations in driving patterns both within and between clusters. To explain the model outcomes, we plot the actual and recommended results for vehicle attributes. This approach helps identify clusters where the model performs well and where it struggles, allowing us to pinpoint low- and high-performance trajectories. Such analysis not only highlights the model’s strengths and weaknesses across different segments but also provides insights into areas where its recommendations are less accurate. This enables further refinement of the model, improving both its reliability and interpretability.

Moreover, time series visualizations for the best- and worst-performing trajectories (Fig 4c) enable us to track and compare driving behaviors at each time step, helping to identify patterns, trends, and discrepancies between observed and recommended actions. Additionally, within each identified cluster, we perform a comparative analysis of low- and high-performance trajectories across each time step (Fig 4d). This comparison highlights variations in model accuracy, making it easier to identify where and why the model exhibits performance differences over time and how these discrepancies can be explained by changes in attributes. Such temporal analysis offers valuable insights into the dynamic nature of driving behaviors and the model’s performance across many different input scenarios.

Collectively, these visual-based analyses contribute to a holistic understanding of the model’s performance by facilitating comprehensive exploration of how different clusters behave in relation to their predicted and actual outcomes. By visually representing the data, we can identify trends, patterns, and anomalies within specific clusters that might not be apparent through quantitative metrics alone. This enables a nuanced evaluation of trajectory- or cluster-specific attributes, allowing researchers to assess not only overall prediction accuracy but also the underlying factors influencing performance. For example, visualizations can reveal which attributes are most strongly associated with successful recommendations in certain clusters while highlighting areas of inconsistency or error. It also assists researchers in identifying why certain trajectories deviate from the patterns exhibited by similar trajectories within the same cluster. By examining these discrepancies, researchers can gain insight into the unique factors or behaviors that contribute to such variations. This analysis is crucial for understanding the complexity of driving behavior, including abrupt changes in speed, uncharacteristic maneuvers, or responses to unexpected environmental conditions. This deeper insight is crucial for refining the model, as it informs targeted improvements and enhances the interpretability of the results, ultimately contributing to more effective decision-making in driving behavior analysis and road safety strategies.

### 3.2 Implementation details

The implementation of the outlined methodology involves several specialized tools and frameworks. Python serves as the primary platform for data preprocessing, leveraging essential libraries, such as Pandas, for efficient data manipulation and Matplotlib for visualization of results. In the modeling phase, a supervised Long Short-Term Memory (LSTM) network is developed using Keras, a prominent deep-learning framework, to capture and predict the sequence of state transitions over time. Additionally, K-means clustering is used for the grouping of trajectories based on their attributes, systematically organizing driving patterns according to computed, multidimensional reward metrics. This clustering approach not only facilitates the identification of distinct trajectory behaviors but also supports the development of a predictive model that enables the classification of new trajectories based on their associated rewards.

## 4. Case study

### 4.1 Data preprocessing

#### 4.1.1 Data overview.

**Fig 5:**
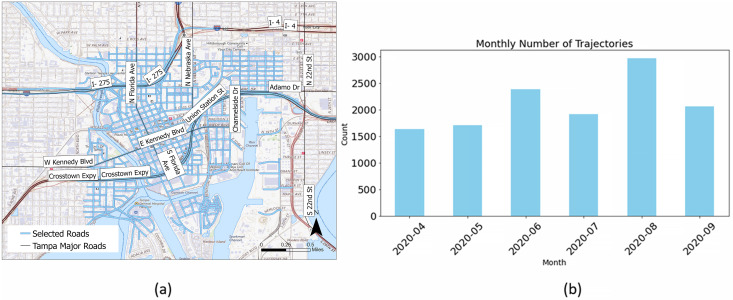
(a) Distribution of roads utilized for collecting Basic Safety Message (BSM) data along with (b) the total monthly count of trajectories recorded.(Base map source: USGS National Map (US Topo). Public domain data courtesy of the U.S. Geological Survey.)

To demonstrate our model performance, we use connected vehicle (CV) data collected in Tampa, Florida, from April to September 2020 (Fig 5). In September 2015, the Tampa Hillsborough Expressway Authority (THEA) partnered with the U.S. Department of Transportation to launch a CV pilot program. The data for this study were sourced from THEA’s daily, near-real-time publications. These datasets are publicly available through the U.S. DOT Connected Vehicle Pilot Deployment Program Data Portal (see Data Availability Statement for details). THEA’s project generated three types of datasets: Basic Safety Messages (BSM), Signal Phasing and Timing (SPaT) messages, and Traveler Information Messages (TIM). As mentioned above, the BSM data were collected from vehicles equipped with sensors that provided detailed information on vehicle status, location, and a sequence of historical positions with corresponding timestamps. By leveraging this historical tracking information, we reconstruct recent vehicle trajectories, enabling analysis of past speeds, acceleration or deceleration, and direction of movement. On average, about 750 distinct trajectories per month were selected for analysis (based on the criteria that each trajectory should have a total time span of at least 30 seconds with a minimum of 60 points to ensure sufficient data for reconstruction and representation). The selected road network in Tampa includes 932 unique road segments.

#### 4.1.2 Trajectory reconstruction.

**Fig 6:**
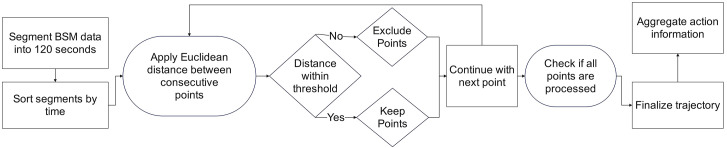
Trajectory reconstruction workflow.

The reconstruction of trajectories (Fig 6) involves dividing the dataset into more manageable units by segmenting the tracked point records from hourly BSM data into 120-second intervals with a distance threshold of 25 meters. Once the dataset is restructured, each segment is sorted by time to preserve the temporal sequence of events and to maintain the integrity of the temporal dynamics within the trajectory. A distance-based filtering method is then applied. Starting from the initial tracking point, the Euclidean distance between consecutive points is calculated. Predefined speed and distance thresholds are used to determine which points will be included in the analysis—if, for instance, the data suggest that a vehicle covered 120 meters in a short time interval, we consider that to be an impossibility, and those points are excluded to mitigate noise and reduce outliers. Only points within the acceptable thresholds are retained. Once a trajectory is complete, the process moves on to the next tracking point that is not yet associated with any existing trajectory. This iterative process continues until all points are processed, generating discrete trajectories. To compare the model’s performance under different levels of temporal granularity, we performed trajectory aggregations at 0.5-second, 1-second, and 3-second intervals. This multi-scale approach allowed us to determine the optimal time frame for summarizing the trajectory’s key temporal features and demonstrated the model’s sensitivity to varying data aggregation periods. This step serves as the finalization phase of the trajectory reconstruction process since aggregating actions over short intervals provides a consolidated view of the trajectory’s key characteristics. Moreover, this aggregated information contributes to the formation of the final trajectory, offering a comprehensive representation of actions taken during specific time segments. The finalized trajectories, enriched with the averaged action information, form the output of the data processing step (see S1 Fig for a data preprocessing flowchart).

### 4.2 Model training and evaluation

The model configuration process involves identifying hyperparameters, architecture choices, and training strategies to determine the optimal settings. We perform clustering prior to applying the LSTM in order to group trajectories based on speed and average actions (e.g., lateral and longitudinal movements). For each identified cluster, we apply LSTM learning within the cluster. The hidden dimensions for the LSTM model are selected based on a combination of empirical testing and commonly accepted practices in the field. Parameters are fine-tuned through cross-validation to optimize performance on the dataset. Specifically, the aim is to balance the model’s capacity to learn complex patterns with the risk of overfitting—in other words, choosing hidden dimensions that provide the best trade-offs and improve separation in the clustering stage. In our study, the major machine learning parameters for the feature extraction are the hidden dimensions used in the LSTM layer. A series of candidate dimension numbers based on insights from existing research [36–39] informed our parameter selection—particularly those related to the hidden dimensions used in neural networks. After initial tests of the LSTM-based clustering, the model was configured with a hidden layer dimension of 64 and trained over 800 epochs, utilizing the tanh activation function and mean squared error as the loss function. These settings are designed to effectively capture the temporal dependencies in the trajectory data while minimizing prediction errors. By integrating attribute-based clustering with LSTM-driven feature extraction, this method enhances trajectory pattern analysis, yielding more accurate and interpretable results. The models are then evaluated through a comparison of actual and predicted driving outcomes. A detailed breakdown of the speed categorization and range definitions utilized for model evaluation can be found in S3 Text and S2 Figure.

**Fig 7:**
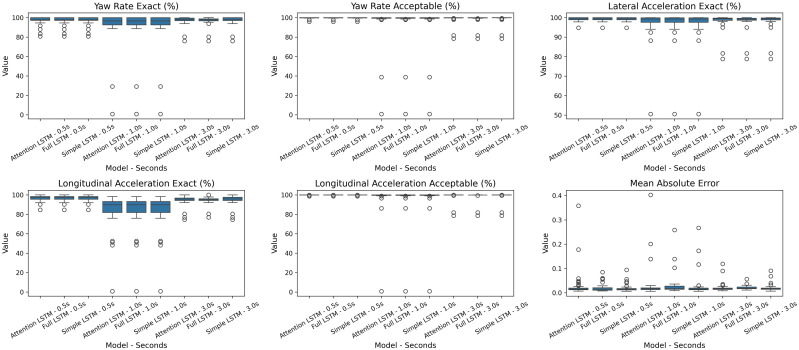
Model performance results with different temporal aggregations.

To better assess our model performance, we included a comparative analysis against different model architectures and prediction horizons to thoroughly evaluate our approach. We chose to compare our proposed LSTM model, which is an Attention LSTM [40]—an LSTM augmented with an attention mechanism that enables the model to focus on the most relevant time steps when making predictions—with both simple LSTM and full LSTM models to establish a baseline and demonstrate the specific value of our architectural innovations. The Simple LSTM, which lacks an attention mechanism, serves as a fundamental benchmark, while the Full LSTM represents a more traditional, comprehensive approach. Similarly, evaluating the models at different time steps (0.5, 1, and 3 seconds) is critical for understanding their practical utility (see model configurations in S3 Text). This range of prediction horizons allows us to assess the model’s ability to forecast actions across different temporal contexts, thereby better representing real-world V2X applications. To evaluate prediction quality, we define two levels of accuracy: an Exact prediction indicates that the forecasted action falls within the same predefined action range as the observed ground truth, while an Acceptable prediction allows for one adjacent range of tolerance, reflecting that small deviations are often behaviorally insignificant. The specific action ranges (for acceleration, deceleration, and yaw rate) are detailed in S1 Fig. Based on these criteria, the Attention LSTM demonstrates more consistent predictive accuracy. As shown in Fig 7, the model achieves a higher proportion of predictions within the exact and acceptable categories. This performance results suggest that the attention LSTM mechanism improves temporal representation by dynamically weighing important segments of the time-series data, enabling the model to prioritize salient features and subtle changes for more precise action prediction.

### 4.3 Action analysis

#### 4.3.1 Cluster-based analysis.

a Clustering Results

**Fig 8:**
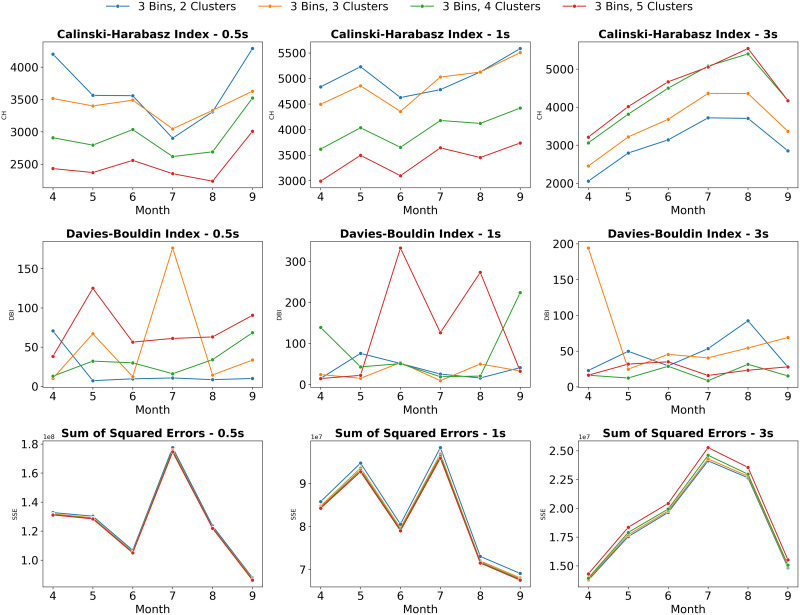
Clustering results comparison across different total numbers of clusters.

The comparison of clustering results shows that using speed bins significantly aids in clustering and predicting driving patterns, particularly with three speed bins, which enhances the grouping of trajectories into distinct categories. The optimal configuration was found to be nine clusters, achieved by grouping trajectories into three speed bins, with each bin further divided into three clusters based on lateral acceleration, longitudinal acceleration, and yaw rate. This approach involves initially grouping trajectories by speed and then applying K-means clustering within each speed bin. For example, in Fig 8, the clustering results are evaluated using three key metrics: the Calinski-Harabasz Index (CH) for measuring how well-defined and separated the clusters are, the Davies-Bouldin Index (DBI) for evaluating the similarity between each cluster, and the Sum of Squared Errors (SSE) for measuring a cluster’s compactness. Across all months and trajectory groups, the configuration that classifies trajectories into 3 speed bins with 3 clusters each generally performs the best. It consistently shows a significantly higher average CH index and a notably lower average DBI compared to other groups. Additionally, it consistently demonstrates the lowest SSE values, which indicates that its clusters are more compact. These results suggest that this configuration allows the clustering algorithm to generate more well-separated and compact groups, indicating a superior grouping of similar driving behaviors.

**Fig 9:**
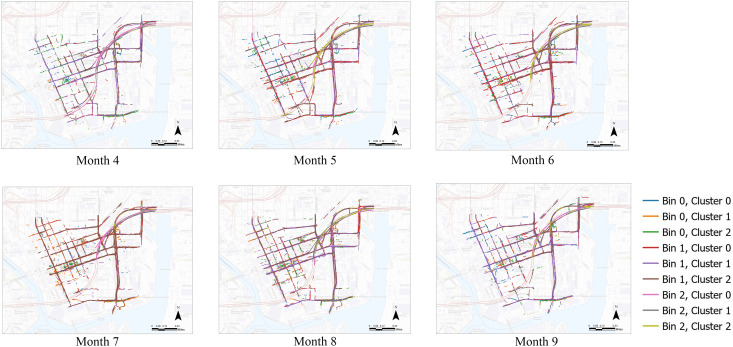
Distribution of clusters by month. (Base map source: USGS National Map (US Topo). Public domain data courtesy of the U.S. Geological Survey.).

Fig 9 visualizes the monthly distribution of driving attributes from Month 4 to Month 9. In this visual representation, highway road segments usually produce more consistent results. Downtown areas show more variation—illustrated in this image by the variety of road segment colors—possibly due to events, holidays, or generally less predictable driving conditions in these areas. These visual patterns are consistent with the findings from our clustering analysis. We also observe that yaw rates are consistently low across all months, potentially indicating that vehicles mostly travel in straight lines or along gentle curves. This is consistent with the visual presentation of this section of the Tampa road system, which features many straightaways, slightly curved highways, and few sharp turns. Other patterns revealed by the cluster analysis—such as a slight decrease in yaw rates over the months studied or month-to-month fluctuations in lateral acceleration—are more difficult to see in this map view, and other visualization tools (such as those presented in the next section) may be better suited for illustrating these changes.

b Cluster status

**Fig 10:**
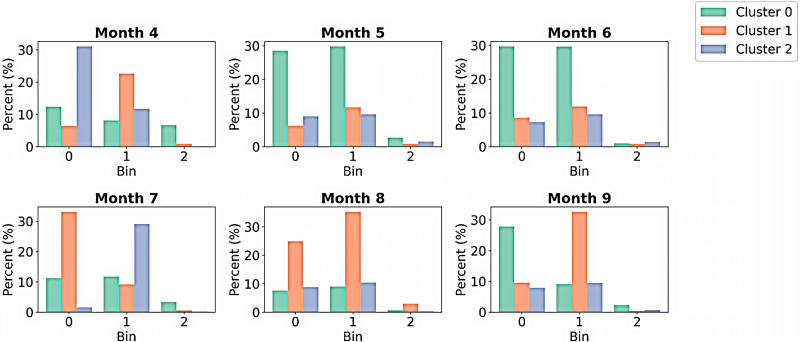
Percentage distribution of monthly trajectories across nine clusters: three speed bins, each containing three clusters.

To provide better insights into the within-cluster performance analysis, a series of visualization techniques is applied in addition to the simple line plots that demonstrate the overall model performance. Fig 10 illustrates the percentage of trajectories that fall into the nine total clusters (grouped into three speed bins, with three clusters per bin). For example, Bin 0 (low speeds) generally exhibits higher percentages across the months than other bins. This trend indicates a prevalence of low-speed trajectories during these months. In contrast, Bin 1 (moderate speeds) displays more variability in its percentages but has high values in Months 8 and 9, suggesting a higher proportion of moderate-speed driving behaviors during these periods. Conversely, Bin 2 (high speeds) shows generally lower percentages during the whole study period, indicating that fewer trajectories overall fall within the high speed range.

**Fig 11:**
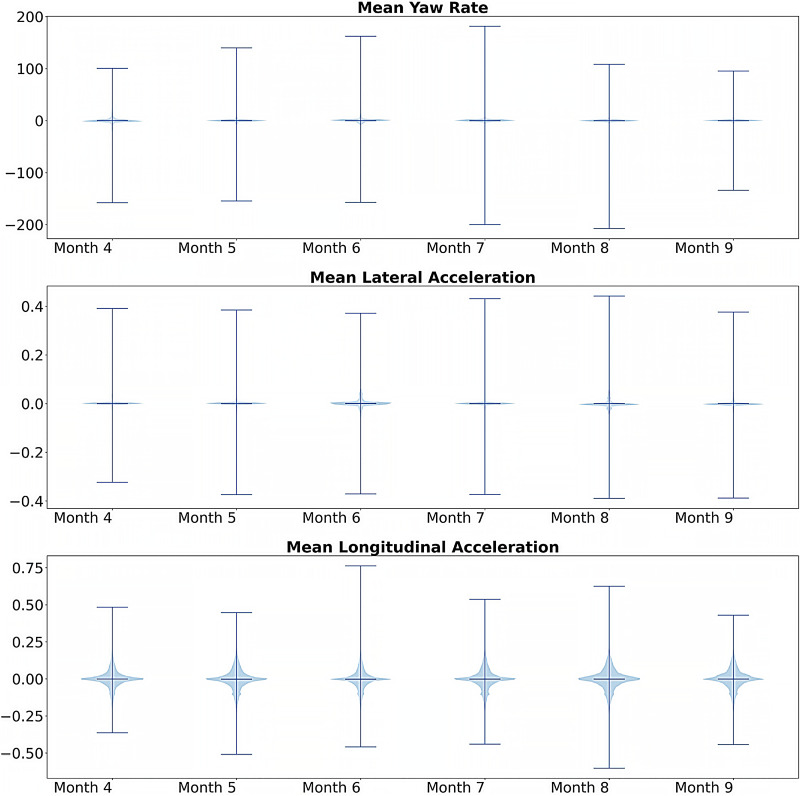
Violin plots showing key attribute distributions by month.

To further explore variation between and within clusters, Fig 11 presents month-to-month comparisons that can reveal patterns across the bins and clusters. mean yaw rate, mean lateral acceleration, and mean longitudinal acceleration from Month 4 to Month 9. The mean yaw rate shows the highest variability, with a wide distribution and long whiskers, suggesting frequent and large rotational movements, while the mean lateral acceleration is the most stable, with a very narrow distribution tightly clustered around zero. The mean longitudinal acceleration shows moderate variability and a slight asymmetry, with a wider spread toward negative values, potentially indicating more frequent or varied braking events. Despite these differences in variability, all three metrics show a generally consistent pattern across the months, with no clear trend of increasing or decreasing stability over time.

**Fig 12:**
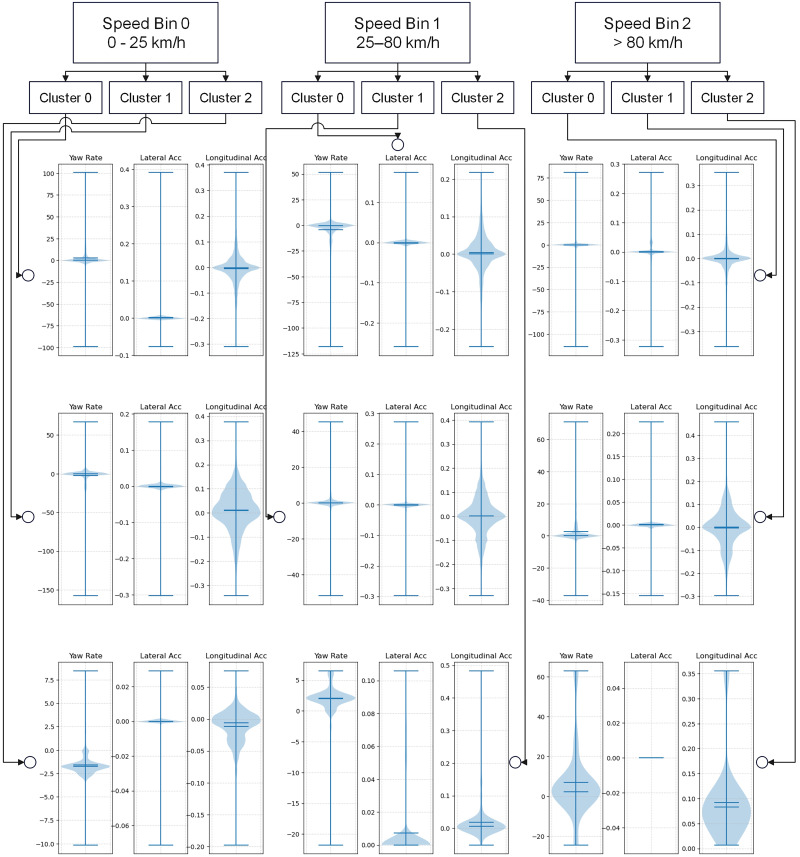
Tree plot showing the distribution of attributes for clusters within each speed bin for Month 4.

To further enhance the visualization of attribute pattern changes, we utilize the boxplots in Fig 12 to develop a hierarchical view, creating a tree plot that shows the distribution of attributes for clusters within each speed bin. This tree plot provides both vertical and horizontal perspectives of trajectory patterns. For example, from Bin 0 to Bin 2, the some of the distributions (e.g., longitudinal acceleration) generally exhibit a broader range and higher standard deviation, suggesting more varied and potentially extreme driving behaviors. Additionally, the yaw rate distribution is quite broad, particularly for Cluster 0, suggesting frequent or sharp low-speed turns, like navigating city streets or parking lots. In contrast, the yaw rate distributions for the higher speed bins are less centered around zero and show greater variance, possibly reflecting steering corrections or lane changes at higher speeds. Within each speed bin, Cluster 2 consistently displays more variability, which could signify a wider range of driving styles or behaviors within that cluster.

**Fig 13:**
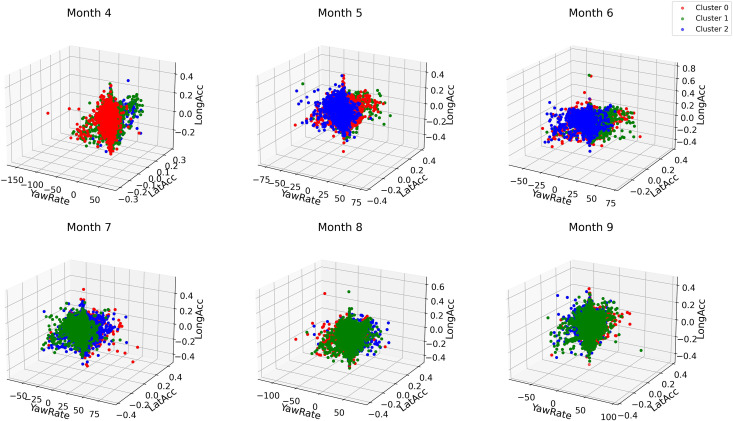
Example of a 3D scatter plot showing attribute values within different clusters for Speed Bin 1 data.

To facilitate the exploration of data distribution for actual driving behaviors, we propose a series of 3D scatter plots for the attribute values, which reinforces the information provided by the tree plots and map views above. For example, Fig 13 illustrates the distribution of all values of actual yaw rates, lateral acceleration, and longitudinal acceleration across different clusters in Speed Bin 1. While matrices like the silhouette score provide a broad view of model performance, they offer limited insights on a finer scale, which these 3D plots improve upon. To demonstrate, in Speed Bin 1, the data points show increased errors and disparities due to a wider spread in Months 4, 5, and 8, revealing a notable variation in lateral acceleration. This variation contributes to the larger discrepancies observed during these months, with a larger spread and visible outliers evident in the data. This pattern is easier to identify on these 3D plots than in the previous visualizations.

c Trajectory-based pattern analysis

To investigate driving behavior, it is also essential to move beyond the patterns observed at the cluster level since finer variations or drastic changes in individual trajectories within clusters can equate to unsafe or unexpected outcomes on the road. Therefore, we propose several visualization techniques for trajectory-based pattern analysis to effectively capture these details. To test the driving behavior model’s ability to detect outliers or uncommon driving patterns, we built a matrix to evaluate how well the model’s recommended driving actions align with real-world data.

**Fig 14:**
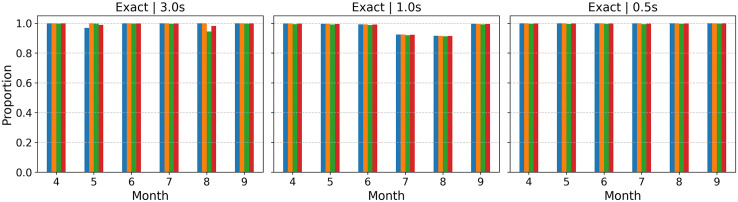
Heatmaps of the matrix showing the trajectories based on values in the predicted and actual ranges for all months.

Based on the Fig 14, the proportion of accurately predicted categories shows that the model performs exceptionally well across all metrics, including Lateral Acceleration, Longitudinal Acceleration, and Yaw Rate, as well as the Overall Rate. The proportion values for each of these metrics are consistently at or very close to 1.0, which indicates that the model’s predictions align almost perfectly with the actual results for the same category outcomes. This high level of accuracy is maintained across different time horizons (3.0s, 1.0s, and 0.5s) and across the months shown (4–9). The consistency of these results demonstrates that the model is accurate, reliable, and stable, as there are no significant fluctuations in performance over time or with changes in the different prediction windows.

**Fig 15:**
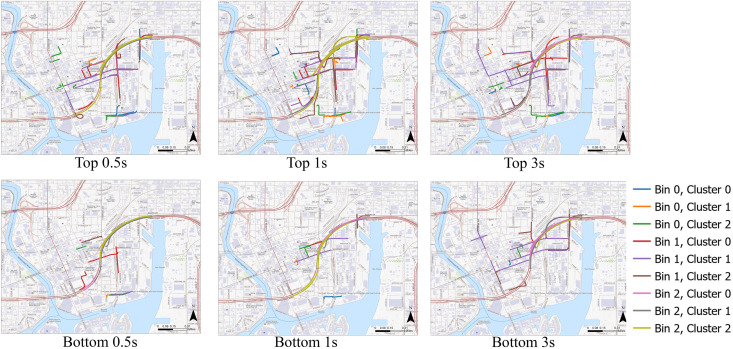
Distribution of the top 10 and bottom 10 trajectories identified through a trajectory-level analysis. Base map source: USGS National Map (US Topo). Public domain data courtesy of the U.S. Geological Survey.

**Fig 16:**
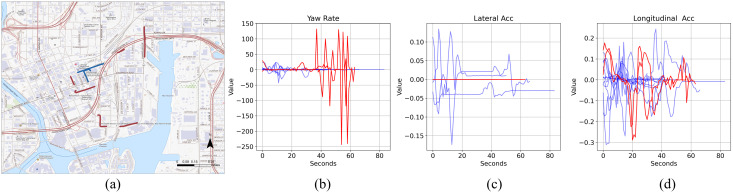
The 10 trajectories with the highest prediction accuracy (blue) and the 10 with the lowest accuracy (red) for specified attributes. Panels (b-d) are time-series plots illustrating individual trajectories alongside their respective attributes. The plots are based on data from Month 4, Speed Bin 0, and Cluster 2. Base map source: USGS National Map (US Topo). Public domain data courtesy of the U.S. Geological Survey.

In Figs 15 and 16, we continue trajectory-level analysis by identifying the top 10 and bottom 10 trajectories based on model performance, illustrating the distinctions between low-performing and high-performing clusters using Month 4 data. The plots indicate that low-performing trajectories can sometimes demonstrate less variation overall, but when there is variation, those changes are sudden and drastic, such as the abrupt change in yaw rate shown in Fig 16. Having a few sudden changes amid otherwise stable data complicates the model’s ability to characterize such behaviors, thereby making it challenging to generalize or categorize the trajectories accurately into the appropriate cluster groups. Furthermore, these low-performing trajectories do not show significant patterns in spatial distribution and are found across various parts of the study area. In contrast, high-performing trajectories display some spatial clustering—for example, several of them cluster around highways. This suggests that trajectories in these areas are more easily grouped, and their driving behaviors can be predicted more reliably.

d Safety implications

Determining whether common actions are inherently safer than outlier behaviors presents a nuanced challenge, particularly when analyzing complex driving patterns that lack clear classification criteria. Identifying common behaviors through unsupervised clustering can help generalize driving patterns, especially when guidelines for classification are unclear, enabling automatic and efficient clustering. Visualizing these patterns within and between clusters plays a crucial role in enhancing model transparency, helping researchers understand why specific behaviors are grouped together and making the AI model more explainable. However, driving behavior is inherently complex, and outliers or sudden, drastic changes in behavior can still occur within clusters. These outliers may follow general patterns but display subtle variations or quick shifts that pose significant risks on the road. Differentiating such behaviors requires both aggregated and trajectory-level analysis. To mitigate safety risks, visualizations like boxplots are essential for exploring the overall characteristics of clusters as well as for investigating why certain trajectories deviate from expected norms. Even in seemingly stable or safe clusters, small anomalies may represent risks. This is where easily explainable AI models, enhanced by visualization, become especially valuable. Combining more granular data and visualization tools makes it easier to accurately identify true outliers and differentiate between safe and risky driving behaviors, ensuring a more informed and transparent analytical process.

### 4.5 Discussion and summary

Through this case study, we have demonstrated the application of our hierarchical, action-learning model using V2X data from Tampa, underscoring the importance of analyzing driving behavior through a structured framework. Our approach is designed to effectively utilize existing V2X data infrastructure by developing a simple and effective solution for capturing both individual vehicles and surrounding driving data. The inclusion of hierarchical clustering provides an interpretable framework so that it is easier to explore and analyze the resulting behavioral patterns. The use of an LSTM framework is also important for modeling the temporal variations and dependencies inherent in vehicle trajectories, allowing for a more accurate and robust analysis. Furthermore, the flexibility and consistent performance of our model demonstrate robust model transferability, and the modular nature of our pipeline ensures it can be potentially improved with other advanced models in the future.

Through this systematic framework, the hierarchical model captures between-cluster and within-cluster variations, revealing nuanced behavioral patterns that are often overlooked by simpler models. Additionally, this study emphasizes the value of developing explainable AI models enhanced by visualization tools, which collectively make the dynamics of driving behavior more transparent and interpretable. Driving behavior is inherently complex, with continuous changes influenced by factors like speed, road conditions, and environmental variables. Unlike other domains with well-defined classification thresholds, driving patterns lack such clarity. As a result, trajectories exhibit varying levels of risk, sometimes appearing generally safe while containing short bursts of drastic changes that can increase potential danger. This complexity makes explainable AI models critical, as they not only help model these dynamic behaviors but also render them understandable for stakeholders, including transportation engineers, policymakers, and individual drivers who could receive real-time safety messages.

Hierarchical clustering is particularly effective in revealing broad patterns and capturing hidden variations in driving behavior. By using speed as the first key factor in this hierarchical approach, we can group driving behaviors based on their context. For example, drivers navigating different speed limits or adjusting to changing road conditions experience varying levels of risk and exhibit varying behavioral shifts in response. Organizing data into speed bins allows for a more detailed analysis of these patterns, reflecting real-world scenarios where drivers must continuously adapt to environmental factors like weather or traffic. This level of granularity then enables the identification and comparison of behaviors within defined speed contexts, providing a clearer view of overall risk. In addition, various visualization techniques—such as box plots, time-sequence plots, and scatter plots of hidden dimensions and driver attributes—are valuable tools for making the model’s decision-making process more interpretable. These visual aids help to explain the model’s results and allow for deeper investigation into abnormal behaviors, outliers, or sudden shifts that could increase driving risk. Box plots in particular highlight how different clusters contain a variety of driving behaviors, while time-sequence plots trace the evolution of these behaviors over time. By visualizing the model’s decision-making process, we can better understand which clusters pose higher risks, which can then aid in the identification of specific factors contributing to these risks.

Through real-world scenarios represented in the Tampa V2X data, we demonstrate that our explainable, hierarchical, action-learning approach is not only theoretically robust but also highly applicable to everyday driving. To implement fine-grained behavioral modeling and visualization, we proposed, executed, and evaluated a comprehensive workflow using critical variables from BSM data derived from six months of a CV pilot study in Florida. Our model effectively captures how drivers adjust their behavior in response to dynamic road conditions, showing that clustering based on speed categories is a valuable strategy for analyzing driving data. By integrating visualization tools, the AI model becomes more explainable and actionable, contributing to both safer driving systems and deeper insights into the behaviors that elevate driving risk.

While the V2X technology and models presented in this research have shown promising results, several limitations still exist. Due to current V2X data limitations, our model was developed using a limited set of driving information. While incorporating additional features like weather and traffic could further enhance performance, our approach was intentionally designed to be effective with minimal data to fully utilize the current V2X data. This makes it a more practical and transferable solution for real-world scenarios where rich environmental data may be unavailable or difficult to acquire. Moreover, the model’s modular design allows it to be easily updated with new data features and more advanced algorithms as they become accessible and computationally feasible, ensuring its future scalability and effectiveness. Currently, V2X technology is still not yet widely deployed, and many vehicles still lack the capability to transmit and receive driving data. As more data become available across different driving locations and conditions, there is great potential for refining and improving the accuracy and reliability of driving behavior analyses. In turn, this will enable the delivery of more precise safety recommendations that can be integrated into transportation plans, added to driver training courses, and even delivered to individual drivers in real time, ultimately enhancing road safety and reducing the occurrence of dangerous driving events.

## 5. Conclusion

With the widespread advancement and deployment of V2X technology, there is immense potential to explore the vast and diverse transportation data generated by connected vehicles and smart transportation infrastructure, leading to safer transportation systems and widespread societal benefits. These vehicle data provide the most comprehensive and standardized record of real-world driving behavior to date, addressing many limitations of prior transportation research, such as the lack of real-time information, high data collection costs, and data inaccuracies. Moreover, V2X data can be integrated with additional key contextual factors, such as environmental conditions, allowing researchers and practitioners to conduct richer analyses and to develop more effective and realistic transportation management strategies. In this research, we introduced a hierarchical, action-learning model that leverages V2X data alongside tailored visualizations, facilitating efficient data mining and enhancing model explainability by offering deeper insights into driving behaviors and risk profiles. By integrating machine learning models with explainable AI techniques, particularly through visualization, we have illustrated how patterns and outliers within clusters can be interpreted with greater clarity. This approach is vital in addressing the complexities of driving behavior, in which actions that deviate from general trends can indicate increased driving risk.

The model’s performance results demonstrate significant promise for future applications, particularly with the increasing accessibility and comprehensiveness of V2X technology. Our model has the potential to be integrated into real-time V2X support systems. With further engineering, this could be achieved by optimizing its computational efficiency and developing specific safety intervention algorithms. For example, we’ve demonstrated that the model is capable of supporting the V2X system to identify potential conflicts by predicting a driver’s intended actions and identifying any variations by comparing vehicles’ movements. Moreover, this capability could also be used by autonomous or semi-autonomous vehicles to anticipate human driver behavior, leading to smoother and more efficient cooperative maneuvers at intersections or in congested traffic. There are several avenues for improvement in future research: For instance, by integrating advanced sensors, the model could incorporate additional variables, such as weather conditions, road types, and traffic density. Furthermore, developing more comprehensive standards to assess transportation risks is essential, as this would facilitate clearer associations between clusters and varying levels of risk. Addressing current limitations is also critical—particularly the reliance on a single dataset from Tampa’s V2X program, which constrains the model’s generalizability. Access to more diverse datasets—especially those that capture risky driving behaviors—would allow for a more thorough evaluation of the model. Additionally, enhancing the visualization design with interactive features would enable users to customize and explore results according to their specific needs. As AI-based models and clustering techniques continue to evolve, these visualization tools should be refined to reflect new methodologies and to offer more detailed, explainable insights into driving behavior and transportation risks.

## Supporting information

S1 Fig Data preprocessing. (JPG)

S2 Fig Model performance. (TIF)

S1 Text Data and problem definition. (DOCX)

S2 Text Trajectory clustering, similar driving context, and action recommendation. (DOCX)

S3 Text Model configuration and evaluation. (DOCX)
