# Supplementary figures and images for: A visualization-supported, hierarchical, action-learning model for driving behavior in a V2X environment
Source: PLoS One. 2026 Jan 2;21(1):e0336268. doi: 10.1371/journal.pone.0336268 (PMC12758737; doi:10.1371/journal.pone.0336268)

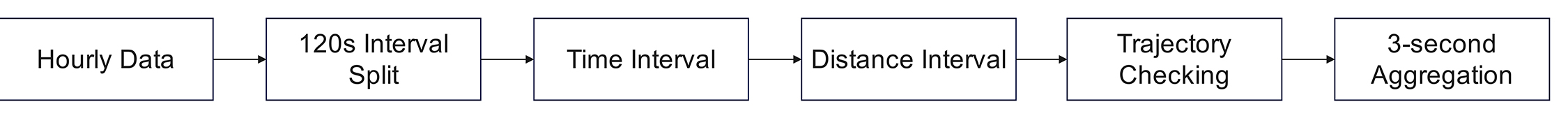

Supplement: S1 Fig — (JPG) [file pone.0336268.s001.jpg]
