# Supplementary material for: A visualization-supported, hierarchical, action-learning model for driving behavior in a V2X environment
Source: PLoS One. 2026 Jan 2;21(1):e0336268. doi: 10.1371/journal.pone.0336268 (PMC12758737; doi:10.1371/journal.pone.0336268)
Supplement: S1 Text — (DOCX) [file pone.0336268.s003.docx]

# **Data and Problem Definition**

We use real-world V2X data—including vehicle position, speed, acceleration, and related factors—to analyze driving behavior. Our study addresses two questions: (1) What are typical driving actions in a given context? (2) How do actual actions deviate from these predictions, and what factors influence the deviations? The following section outlines the formal definitions of the data.

**Definition 1**. Track Point ($p_{i}$): A track point representing a specific location of a vehicle at a given time, including location, speed, yaw rate, acceleration along two directions, and the time difference and distance from the beginning of the vehicle's trajectory. A track point of the vehicle ($p_{i})$describes its status at a time point $t_{i}$:

$p_{i}=(x_{i}, y_{i}, t_{i}, \Delta t_{i}, \Delta d_{i},v_{i} , \tau_{i}, {\alpha_{lat}}_{i}, {\alpha_{lon}}_{i})$ (1)

where:

$x_{i}$ is the longitude value

$y_{i}$ is the latitude value

$t_{i}$ is the timestamp

$\Delta t, \Delta d$ are the time difference and the distance from the beginning of the trajectory to the current location

$v_{i}$ is the vehicle speed

$\tau_{i}$ is the yaw rate, which is the angular rotation of a vehicle around its vertical axis, indicating the sharpness of a turn

$\alpha_{lat}$ is the lateral acceleration (acceleration during a turn), $\alpha_{lon}$ is the longitudinal acceleration (acceleration in a straight line)

**Definition 2.** Trajectory (*tr*): A trajectory is a sequence of track points ($p_{i}$) that describes the movement of a vehicle over a period of time.

$tr=\{p_{i}\}$ (2)

Each track point ($p_{i}$) contains information about the vehicle (see Definition 1) that supports path reconstruction and driving pattern analysis.

**Definition 3.** To capture driving dynamics, we define actions at each trajectory point using lateral acceleration, longitudinal acceleration, and yaw rate, representing the vehicle’s state. Each action is bounded by lower and upper limits to ensure feasible and safe operation.

**Definition 3.a.** Actions ($a$): An action at a time point of a trajectory is defined as:

$a_{i}=(\tau_{i}, {\alpha_{lat}}_{i}, {\alpha_{lon}}_{i})$ (3)

**Definition 3.b.** Action Range ($ar$): Each action type has a defined lower and upper bound. These bounds are specified as follows:

${ar}_{i}=(\tau_{r_{i}}, {\alpha r_{lat}}_{i}, {\alpha r_{lon}}_{i})$ (4)

The table (Table 1) below lists driving actions and their corresponding acceleration ranges, providing a simplified view of typical behaviors. These ranges are used to capture minor fluctuations, support modeling of driving actions, and offer insights into how different behaviors impact overall safety.

Table 1. Action range values (Albaba & Yildiz, 2021; SHRP2 NDS, n.d.)

| **Action** | **Action Range** | **Intervals** |
| --- | --- | --- |
| Maintain | Acceleration is sampled from a normal distribution with μ = 0 m/s^2^ and σ = 0.075 m/s^2^ | N/A |
| Accelerate | Acceleration is sampled from a uniform distribution between 0.5 m/s^2^ and 2.5 m/s^2^ | 0.5, 2.5 m/s^2^ |
| Hard Accelerate | Acceleration is sampled from an inverse half normal distribution with μ = 3.2 m/s^2^ and σ = 0.3 m/s^2^ |  |
| Decelerate | Acceleration is sampled from a uniform distribution between -0.5 m/s^2^ and -2.5m/s^2^ | -2.5, -0.5, -0.075 m/s^2^ |
| Hard Decelerate | Acceleration is sampled from a half normal distribution with μ = -3.2 m/s^2^ and σ = 0.3 m/s^2^ |  |
| Yaw Rate | The yaw rate is defined as 0–360 degrees for right turns and -360 to 0 degrees for left turns. | 15, 45, 90 degrees (right turns) and -90, -45, -15 degrees (left turns) |
