# Supplementary material for: A visualization-supported, hierarchical, action-learning model for driving behavior in a V2X environment
Source: PLoS One. 2026 Jan 2;21(1):e0336268. doi: 10.1371/journal.pone.0336268 (PMC12758737; doi:10.1371/journal.pone.0336268)
Supplement: S2 Text — (DOCX) [file pone.0336268.s004.docx]

# **Trajectory Clustering, Similar Driving Context, and Action Recommendation**

We implemented a two-level clustering process on vehicle trajectory data. First, we group trajectories by their mean speed to categorize them into three pre-defined bins: low, medium, and high (as shown above). This initial step ensures that subsequent clustering is performed on comparable data. Within each speed bin, we applied the K-Means algorithm to cluster trajectories based on the driving dynamics, specifically the mean values of yaw rate, lateral acceleration, and longitudinal acceleration. This creates a set of sub-clusters that represent distinct driving behaviors within each speed range. Finally, this process identifies outliers within each sub-cluster by measuring the distance of each trajectory from its cluster center. The resulting clustered and categorized data is then utilized for further analysis within a machine learning model.

We divided the vehicle speed into three distinct bins including low, medium, and high speeds. This approach ensures replicability and semantic interpretability of the bins. Specifically, low speeds (<7 m/s or <25 km/h) represent congestion or stop-and-go conditions. Medium speeds (7–22 m/s or 25–80 km/h) are characteristic of urban cruising or stable traffic flow, while high speeds (≥22 m/s or ≥80 km/h) correspond to free-flow or highway conditions. To assess prediction results, we further categorized both predicted and actual actions into predefined bins to evaluate whether the model captures intended driving behavior rather than relying solely on numerical accuracy. For acceleration, the positive ranges are defined as 0–0.5, 0.5–2.5, and 2.5–3.2 m/s², while the negative ranges (deceleration) are –3.2 to –2.5, –2.5 to –0.5, and –0.5 to 0 m/s². Similarly, yaw rate bins are set at –360 to –90, –90 to –45, –45 to –15, –15 to 0, 0 to 15, 15 to 45, 45 to 90, and 90 to 360 degrees per second. Within this framework, two levels of accuracy are defined: (1) Exact, where a prediction falls into the same predefined action bin as the observed ground truth (e.g., both observed and predicted accelerations are in the 0.5–2.5 m/s² bin); and (2) Acceptable, where a prediction falls into an adjacent bin to the observed ground truth (e.g., the ground truth is 0.5–2.5 m/s², but the prediction is in 0–0.5 or 2.5–3.2 m/s²), reflecting that minor deviations across bins are often behaviorally insignificant.

**Definition 4. Level 1 Clustering:** Speed-based Binning: Define a speed-based clustering function $C_{\text{v }}:T\to\{1,2,\ldots,k_{l1}\}$, where *v* indicates a particular speed bin, and $k$ represents the total number of Level 1 (*l*1) speed bins. Trajectories with similar speeds are assigned to the same cluster:

$c_{\text{v }}\left( {tr}_{i} \right)=f_{Lv}(tr_{i})$*, where* $t_{i}\in T$ (5)

**Definition 5. Level 2 Clustering:** Action-based Clustering: For each speed bin $C_{\text{v }}\left( {tr}_{j},a \right)=c_{j}$, where $1\leq j\leq k$, define an action-based clustering function $C_{a}^{(j)}:T\to\left\{ 1,2,\ldots,l_{j} \right\}$, where $l_{j}$ represents the number of clusters within the $j$th speed bin. Trajectories within the same speed bin are further clustered based on other action attributes:

$C_{va}\left( {tr}_{i} \right)=f_{La}(c(tr_{i}))\text{, where }t_{i}\in T$ (6)

**Definition 6. Similar Driving Context:** Given a collection of trajectories *T* = {$tr$_1_, $tr$_2_, ..., $tr$_n_}, where each trajectory $tr$ consists of a sequence of 𝑚 track points, the goal is to assign cluster labels to these trajectories based on their attributes. In this context, n represents the number of trajectories, 𝑚 denotes the number of points in each trajectory, 𝐼 indicates the point sequence within a trajectory, and 𝑗 refers to the trajectory sequence. This clustering task can be formulated as finding a clustering function $f_{L}$: *T→C* to determine the cluster label of a trajectory, where *C* represents the set of cluster labels, *K* signifies the total number of clusters, and *k* indicates one of the clusters (i.e., the *k*th cluster in the sequence). The function maps each trajectory $tr$ consisting of points $\{p_{i}\}$ to a cluster label $c_{k}$, ensuring that trajectories with similar attribute values are grouped into the same cluster, such that all trajectories in the cluster exhibit greater similarity in speed and actions compared to those in different clusters:

$c\left( tr_{j} \right)=f_{L}\left( tr_{j}, T,m,I \right)$ (7)

**Definition 7. Common Action:** Given a set of trajectories *T* = {*tr*_1_, *tr*_2_, ..., *tr*_n_}, the common action can be defined as follows: For a cluster $c_{k}$, let $T_{c_{k}}$ be the set of trajectories assigned to cluster $c_{k}$. The common action $\hat{a_{i}}$ within cluster $c_{k}$ is determined by learning from the actions of all trajectories within the cluster at time point *t_i_*, such that:

$\hat{a_{i}}(tr)$ *_=_* $f_{aggr}$ *(t_i_, for all tr ∈* $T_{c_{k}}$*)*  (8)

Aggregation is a function that computes a representative or common action for the trajectories in the cluster. The nature of the aggregation function ($f_{aggr}$) depends on the specific context and type of action associated with the trajectories (e.g., average action, most frequent action, and uncommon outlier actions that deviate from the general patterns). For example, in our case, we defined outlier actions as those that deviate from or lie outside the common action ranges defined above.
