# Supplementary material for: A visualization-supported, hierarchical, action-learning model for driving behavior in a V2X environment
Source: PLoS One. 2026 Jan 2;21(1):e0336268. doi: 10.1371/journal.pone.0336268 (PMC12758737; doi:10.1371/journal.pone.0336268)
Supplement: S3 Text — (DOCX) [file pone.0336268.s005.docx]

# **Model Configuration and Evaluation**

The LSTM model is assessed using mean absolute error. In general, the yaw rate tends to be noisy, which can make MAE less reliable for accurately assessing prediction accuracy in this context. Given that acceleration and deceleration have a more direct impact on safety-critical scenarios, the MAE calculation is focused primarily on these action variables. As a result, acceleration and deceleration are emphasized in the MAE evaluation to ensure more accurate and robust performance in safety-sensitive applications.

**Definition 8. Mean absolute error (MAE):**

$MAE\left( y,ŷ \right)=\frac{1}{n}\sum_{i=1}^{n} |a_{i}-{ar}_{i}|$ (9)

where:

$y$ represents the observed action

$ŷ$ represents the predicted action

$n$ is the number of observations

In addition to the various LSTM model variants, an Adversarial Inverse Reinforcement Learning (AIRL) model is also included for comparison of model performance. The results demonstrate that the attention-based LSTM model consistently outperforms the other models. See the table below for a summary of the model configurations.

Table 1. Model Configurations

| **Component** | **LSTM Models** | **AIRL** |
| --- | --- | --- |
| **Architectures** | Simple LSTM: A baseline model with two layers of 64 units each.  Full LSTM: A deeper network with five layers to capture more complex temporal patterns. Additional constraints are added based on predicted actions to improve safety.  Attention LSTM: Incorporates a multi-head attention mechanism to enhance temporal and contextual understanding. | Proximal Policy Optimization (PPO): An advanced reinforcement learning algorithm serving as the core generator.  Reward Network: A simplified network designed to compute rewards, with normalization applied to input data. |
| **Loss Function** | Simple / Full LSTM: Weighted MAE loss with lower weighting on yaw rate due to its high variability.  Attention LSTM: Weighted Huber loss, which is less sensitive to large outliers compared to MAE. | Uses an implicit adversarial loss, training the model to generate realistic actions that are difficult for the discriminator to distinguish from real-world behavior. |
| **Optimizer** | Adam optimizer Learning Rate: Small initial values (1e-4 or 1e-3) for stable training. Learning rate is automatically adjusted during training to ensure optimal convergence. | Uses the standard PPO optimizer. |
| **Training Steps** | Trained for a fixed number of epochs (400 or 800) to ensure performance stabilization. | Trained for a very large number of steps (1,200,000) to support learning in complex environments. |
| **Batch Size** | Batch size: 32 | Generator Replay Buffer: Stores past experiences (buffer size: 2048) for training.  Batch Size: 256 |
| **Regularization** | Dropout (0.2): Prevents overfitting by randomly deactivating neurons during training.  Masking: Handles variable-length sequences. | Uses standard regularization settings for PPO. |
| **Evaluation Metrics** | Mean Absolute Error (MAE): Measures the average absolute difference between predicted and actual values. Precision: The proportion of recommended actions that fall within the correct range. | MAE: Computed for all dimensions except yaw rate.  Precision: Based on action categorization bins. |
